# Supplementary material for: Migration and accumulation of bacteria with chemotaxis and chemokinesis
Source: Eur Phys J E Soft Matter. 2021 Mar 15;44(3):32. doi: 10.1140/epje/s10189-021-00009-w (PMC7960630; doi:10.1140/epje/s10189-021-00009-w)
Supplement: Supplementary file 1 — Supplementary material 1 (pdf 439 KB) [file 10189_2021_9_MOESM1_ESM.pdf]

Supplementary Material for "Migration and accumulation of bacteria with chemotaxis and chemokinesis"

Theresa Jakuszeit,[1,*] James Lindsey-Jones,[1] François J. Peaudecerf,[2] and Ottavio A. Croze[1,†]

[1] *Cavendish Laboratory, University of Cambridge, Cambridge CB3 0HE, U.K.*

[2] *Institute of Environmental Engineering, Department of Civil, Environmental and Geomatic Engineering, ETH Zürich, 8093 Zürich, Switzerland*

ADDITIONAL SIMULATIONS

We present further simulation results for the scenarios described in the manuscript by varying crucial and unknown parameters determining the bacterial response. As the chemokinetic response function is unknown, these include the strength of the chemokinetic speed increase η, the half-saturation concentration of the chemokinetic response ω and the Hill factor n as well as the strength of the chemotactic response δ_0.

Steady linear gradient

The effect of modifying the chemotactic sensitivity δ_0 and the chemokinetic parameters η and ω is shown in Figure S1.

Self-generated gradient

The effect of varying the Hill parameter n of the chemokinetic function $V(C) = 1 + \dfrac{\eta C^n}{C^n + \omega^n}$ is shown in Figure S2(a). Figure S2(b) illustrates the effect of the chemotactic sensitivity δ_0 and the chemokinetic parameters ω and η.

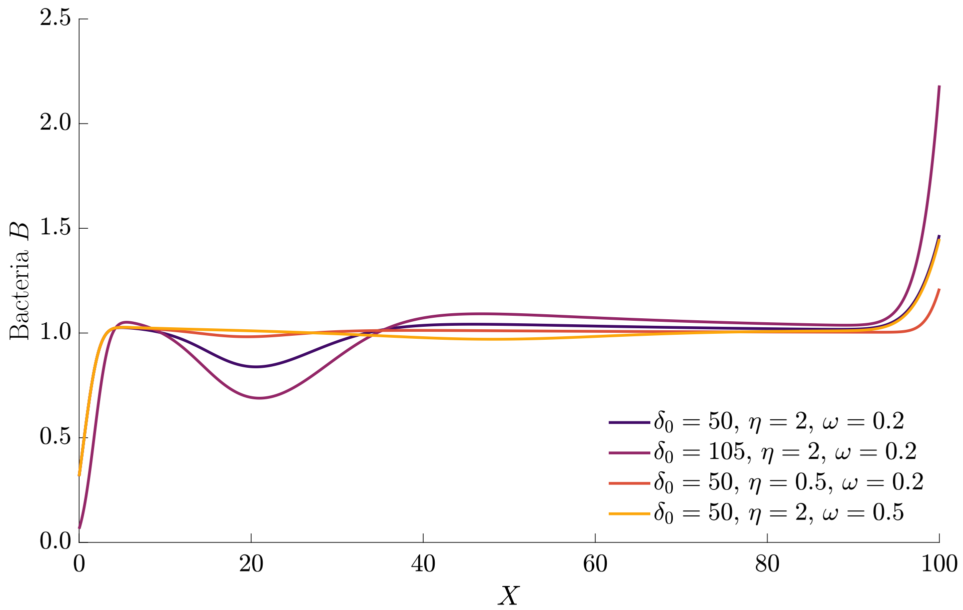

FIG. S1: Bacterial response to a fixed linear attractant gradient as in Fig 3 in the main paper but with varying parameters ($T = 1$).

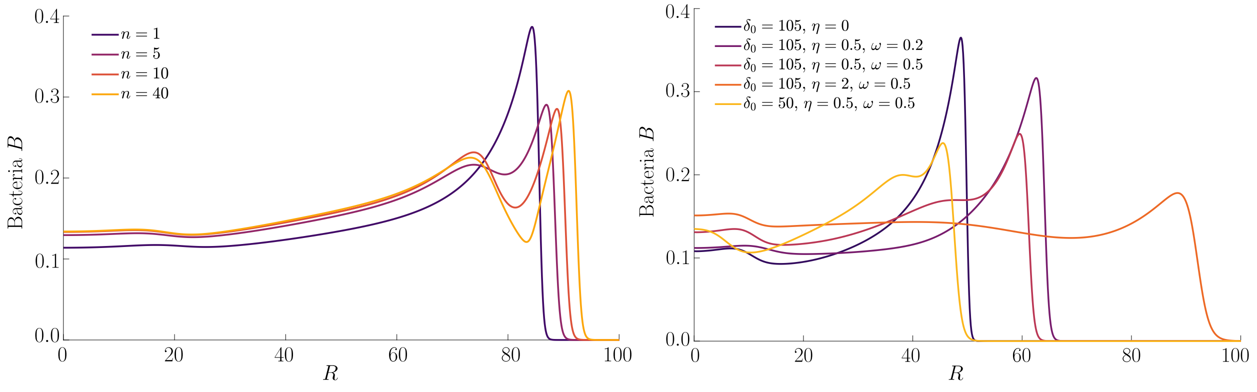

(a) $T = 16.4$ (b) $T = 11.4$

FIG. S2: Bacterial response to a self-generated attractant gradient as in Fig. 5 in the main text but with varying parameters. (a) Varying the Hill parameter in the chemokinetic response function changes the bacterial density at $T = 16.4$ (corresponds to the last column in Fig. 5 in the main text). (b) Varying chemotactic and chemokinetic parameters δ_0, η and ω at $T = 11.4$, where a simulation using the parameters from the main paper is included as a comparison for this time step.

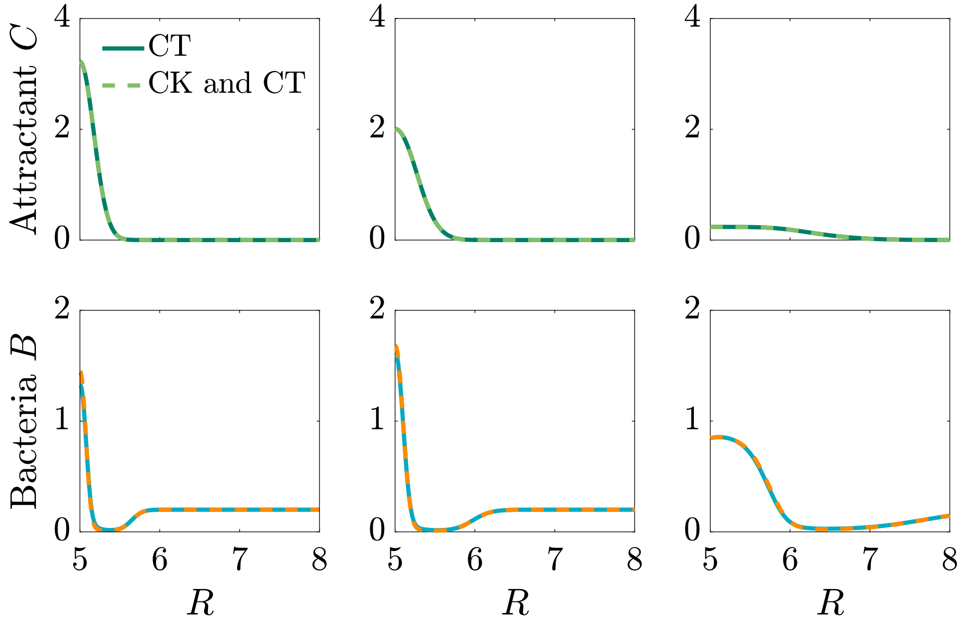

FIG. S3: Diffusing attractant from a transient source. Bacterial populations (bottom row) are attracted to source of diffusing attractant (top row). The chemokinetic-chemotactic population (orange curve) shows a faster and stronger accumulation than the purely chemotactic population (blue curve). Parameters $H = 3.5$, $K_S = 1$, $N = 0.5$, $K_\chi = 0.53$, $\delta_0 = 50$, $\eta = 0.5$, $\omega = 0.5$, $n = 1$, $T = 0.01, 0.05, 0.64$; no bacterial growth.

Transient source

The parameters used in the main text are the same as those used for the steady linear attractant gradient. The results of a simulation that uses the parameters of the self-generated attractant gradient instead are shown in Figure S3.
